# Generalized Steffensen’s inequality by Lidstone interpolation and Montogomery’s identity

**DOI:** 10.1186/s13660-018-1826-4

**Published:** 2018-09-12

**Authors:** Asfand Fahad, Josip Pečarić, Muhammad Imran Qureshi

**Affiliations:** 1 Department of Mathematics, COMSATS University Islamabad, Vehari Campus, Pakistan; 2 Faculty of Textile Technology, University of Zagreb, Zagreb, Croatia; 3 RUDN University, Moscow, Russia

**Keywords:** 26D10, 26D20, Steffensen’s inequality, Green’s function, Montogomery’s identity, $(n+1)$-convex function at a point

## Abstract

By using a Lidstone interpolation, Green’s function and Montogomery’s identity, we prove a new generalization of Steffensen’s inequality. Some related inequalities providing generalizations of certain results given in (J. Math. Inequal. 9(2):481–487, 2015) have also been obtained. Moreover, from these inequalities, we formulate linear functionals and describe their properties.

## Introduction

Steffensen [12] proved the following inequality: if $f,h: [\alpha, \beta] \to\mathbb{R}$, $0\leq h\leq1$, and *f* is decreasing, then

1 $$ \int_{\alpha}^{\beta} f(t) h(t) \,dt \leq \int_{\alpha}^{\alpha+\gamma} f(t) \,dt,\quad \text{where } \gamma= \int_{\alpha}^{\beta} h(t) \,dt. $$

Since then, a generalization and an improvement of Steffensen’s inequality has been a topic of interest of several mathematicians, for example see [10] and the references therein. One generalization of Steffensen’s inequality is given by Pečarić [8].

### Theorem 1

*Consider any non*-*decreasing real valued functions* *g* *on* $[a,b]$ *and* *f* *on interval* *I* (*where* *I* *is such that it contains all* *a*, *b*, $g(a)$ *and* $g(b)$) *such that* *g* *is differentiable*. *Consider the conditions* (i) $g(x)\leq x$ *and* (ii) $g(x)\geq x$.

- *If* (i) *holds*, *then*
  2 $$ \int_{a}^{b} f(t)g'(t) \,dt \leq \int_{g(a)}^{g(b)} f(t) \,dt. $$
- *If* (ii) *holds*, *then inequality in part* (a) *is reversed*.

### Remark 1

In Theorem 1 one may take *g* as an absolutely continuous function instead of differentiable function because if *f* is non-decreasing then the function $F(x)= \int_{a}^{x} f(t) \,dt$ is well defined and $F' = f$ holds almost everywhere on *I*. Then if *g* is any absolutely continuous and non-decreasing function then the substitution $z=g(t)$ in the integral is justified (see [5, Corollary 20.5]), so

3 $$ F \bigl(g(b) \bigr) - F \bigl(g(a) \bigr) = \int_{g(a)}^{g(b)} f(z) \,dz = \int_{a}^{b} f \bigl(g(t) \bigr) g^{(1)}(t) \,dt \leq \int_{a}^{b} f(t) g^{(1)}(t) \,dt, $$

where the last inequality holds when *g* satisfies condition (i).

Recently, Fahad, Pečarić and Praljak proved a generalization [4] of (1) by improving the results given in [8] and [11]. The following is a consequence of a result given in [4].

### Corollary 1

*Consider any non*-*decreasing*, *differentiable and real valued functions* *g* *and* *f* *on* $[a,b]$ *and interval* *I*, *respectively*, (*where* *I* *is such that it contains all* *a*, *b*, $g(a)$ *and* $g(b)$). *If* *f* *is convex*, *then*:

- *If* *g* *satisfies condition* (i) *given in Theorem *1, *then*
  4 $$ f \bigl(g(b) \bigr) \leq f \bigl(g(a) \bigr) + \int_{a}^{b} f^{\prime}(t) g^{\prime}(t) \,dt. $$
- *If* *g* *satisfies condition* (ii) *given in Theorem *1, *then the reverse of the above inequality holds*.

The above corollary gives (3) and consequently Steffensen’s inequality. Now, we present some other consequences from results in [4].

### Corollary 2

*Let* $f:[0,b] \to\mathbb{R}$ *be a differentiable convex function with* $f(0)=0$ *and let* $h:[0,b] \to[0,+\infty)$ *be another function*.

- *If* $\int_{0}^{x} h(t) \,dt \leq x$ *for every* $x\in[0,b]$, *then*
  5 $$ f \biggl( \int_{0}^{b} h(t) \,dt \biggr) \leq \int_{0}^{b} f^{\prime}(t) h(t) \,dt. $$
- *If* $x\leq\int_{0}^{x} h(t) \,dt $ *for every* $x\in[0,b]$, *then the reverse of the above inequality holds*.

### Corollary 3

*Let* *f* *and* *h* *be as in Corollary *2 *and let* $k:[0,b]\to [0,+\infty)$ *and denote* $K(t) = \int_{t}^{b} k(x) \,dx$.

- *If* $\int_{0}^{x} h(t) \,dt \leq x$ *for every* $x\in[0,b]$, *then*
  6 $$ \int_{0}^{b} k(x) f \biggl( \int_{0}^{x} h(t) \,dt \biggr) \,dx \leq \int_{0}^{b} K(t) f^{\prime}(t) h(t) \,dt. $$
- *If* $x\leq\int_{0}^{x} h(t) \,dt $ *for every* $x\in[0,b]$, *then the reverse of the above inequality holds*.

The goal of this paper is to obtain generalized Steffensen’s inequality by proving generalization of (4). Moreover, inequalities (5) and (6) can be used to obtain classical Hardy-type inequalities; see [4]. Keeping in view the importance of (5) and (6) we obtain their generalizations as well. In our construction, we use Green’s function, Montogomery’s identity and Lidstone interpolation. The following lemma is given in [7].

### Lemma 1

*For a function* $f\in C^{2}([a,b])$, *see* [13], *we have*

7 $$\begin{aligned} & f(x)=\frac{b-x}{b-a} f(a)+\frac{x-a}{b-a} f(b)+ \int_{a}^{b} G_{*,1}(x,s) f^{\prime\prime}(s) \,ds, \end{aligned}$$

8 $$\begin{aligned} & f(x)=f(a)+(x-a)f^{\prime}(b)+ \int_{a}^{b} G_{*,2}(x,s) f^{\prime\prime }(s) \,ds, \end{aligned}$$

9 $$\begin{aligned} & f(x)=f(b)+(b-x)f^{\prime}(a)+ \int_{a}^{b} G_{*,3}(x,s) f^{\prime\prime }(s) \,ds, \end{aligned}$$

10 $$\begin{aligned} & f(x)=f(b)-(b-a)f^{\prime}(b)+(x-a)f^{\prime}(a)+ \int_{a}^{b} G_{*,4}(x,s) f^{\prime\prime}(s) \,ds, \end{aligned}$$

11 $$\begin{aligned} & f(x)= f(a)+(b-a)f^{\prime}(a)-(b-x)f^{\prime}(b)+ \int_{a}^{b} G_{*,5}(x,s) f^{\prime\prime}(s) \,ds, \end{aligned}$$

*where*

12 $$\begin{aligned} & G_{*,1}(x,s) = \textstyle\begin{cases} \frac{(x-b)(s-a)}{b-a} & \textit{if } a\leq s \leq x, \\ \frac{(s-b)(x-a)}{b-a} & \textit{if } x< s \leq b,\end{cases}\displaystyle \end{aligned}$$

13 $$\begin{aligned} & G_{*,2}(x,s) = \textstyle\begin{cases} a-s & \textit{if } a\leq s \leq x, \\ a-x & \textit{if } x< s \leq b,\end{cases}\displaystyle \end{aligned}$$

14 $$\begin{aligned} &G_{*,3}(x,s) = \textstyle\begin{cases} x-b & \textit{if } a\leq s \leq x, \\ s-b & \textit{if } x< s \leq b,\end{cases}\displaystyle \end{aligned}$$

15 $$\begin{aligned} & G_{*,4}(x,s) = \textstyle\begin{cases} x-a & \textit{if } a\leq s \leq x, \\ s-a & \textit{if } x< s \leq b,\end{cases}\displaystyle \end{aligned}$$

*and*

16 $$ G_{*,5}(x,s) = \textstyle\begin{cases} b-s & \textit{if } a\leq s \leq x, \\ b-x & \textit{if } x< s \leq b.\end{cases} $$

Consider the following functions on $[a,b]\times[a,b]$:

17 $$\begin{aligned} & p_{1}(x,s) = \textstyle\begin{cases} \frac{s-a}{b-a} & \text{if } a\leq s \leq x, \\ \frac{s-b}{b-a} & \text{if } x< s \leq b,\end{cases}\displaystyle \end{aligned}$$

18 $$\begin{aligned} & p_{2}(x,s) = \textstyle\begin{cases} 0 & \text{if } a\leq s \leq x, \\ -1 & \text{if } x< s \leq b,\end{cases}\displaystyle \end{aligned}$$

19 $$\begin{aligned} & p_{3}(x,s) = \textstyle\begin{cases} 1 & \text{if } a\leq s \leq x, \\ 0 & \text{if } x< s \leq b.\end{cases}\displaystyle \end{aligned}$$

Clearly,

20 $$ \begin{aligned}& p_{i}(x,s)={G_{*,i}}_{x}(x,s)\quad \text{for all }i=1,2,3,\\ & p_{2}(x,s)={G_{*,5}}_{x}(x,s) \quad\mbox{and}\quad p_{3}(x,s)={G_{*,4}}_{x}(x,s). \end{aligned} $$

Throughout the calculations in the main results, we will use $p_{i}(x,s)$ corresponding to ${G_{*,i}}(x,s)$ for $i=1,2,3$ and for $G_{4}(x,s)$, and $G_{5}(x,s)$, $p_{3}(x,s)$ and $p_{2}(x,s)$, respectively.

Now, we consider the following simple lemma.

### Lemma 2

*Let* $f\in C^{1}[a,b]$ *and* $p_{i}(x,s)$, *for* $i=1,2,3$ *be as defined above then*

21 $$\begin{aligned} & f(x)=\frac{1}{b-a} \int_{a}^{b} f(s) \,ds + \int_{a}^{b} p_{1}(x,s) f^{\prime}(s) \,ds, \end{aligned}$$

22 $$\begin{aligned} & f(x)=f(b) + \int_{a}^{b} p_{2}(x,s) f^{\prime}(s) \,ds, \end{aligned}$$

*and*

23 $$ f(x)=f(a) + \int_{a}^{b} p_{3}(x,s) f^{\prime}(s) \,ds. $$

### Proof

For fix $i=1,2,3$ consider

$$\int_{a}^{b} p_{i}(x,s) f^{\prime}(s) \,ds= \int_{a}^{x} p_{i}(x,s) f^{\prime}(s) \,ds+ \int_{x}^{b} p_{i}(x,s) f^{\prime}(s) \,ds. $$

By replacing specific value of $p_{i}(x,s)$ and simplifying we get the required identities. □

We conclude the section by recalling the Lidstone interpolation and some of its properties. The details related to the Lidstone interpolation can be found in [1]. We start with the following lemma given in [1].

### Lemma 3

*If* $f \in C^{(2n)}([0,1])$, *then*

24 $$ f(x) = P_{L} (x) + e_{L} (x) = \sum _{k=0}^{n-1} \bigl[ f^{(2k)} (0) \Lambda_{k} (1-x) +f^{(2k)} (1) \Lambda_{k} (x) \bigr] + \int_{0}^{1} G_{n} (x,s)f^{(2n)} (s) \,ds, $$

*where* $\Lambda_{k}$ *is a Lidstone polynomial*; *see* [1]. *Moreover*,

25 $$ G_{1}(x,s) = G(x,s) = \textstyle\begin{cases} (x-1)s & \textit{if } s \leq x, \\ (s-1)x & \textit{if } x \leq s, \end{cases} $$

*is a homogeneous Green’s function of the differential operator* $\frac {d^{2}}{ds^{2}}$ *on* $[0,1]$, *and with the successive iterates of* $G(x,s)$

26 $$ G_{n}(x,s) = \int_{0}^{1}G_{1}(x,u)G_{n-1}(u,s) \,du,\quad n\geq2. $$

From the lemma it is not difficult to conclude that a function $f \in C^{(2n)}([a,b])$ can be represented by using a Lidstone interpolation in the following way:

27 $$\begin{aligned} f(s)={}& \sum_{i=0}^{n-1} (b-a)^{2i} f^{(2i)} (a) \Lambda_{i} \biggl( \frac {b-s}{b-a} \biggr)+ \sum_{i=0}^{n-1}(b-a)^{2i} f^{(2i)}(b)\Lambda_{i} \biggl(\frac {s-a}{b-a} \biggr) \\ &{}+(b-a)^{2n-1} \int_{a}^{b} G_{n} \biggl( \frac{s-a}{b-a},\frac{\xi -a}{b-a} \biggr)f^{(2n)}(\xi) \,d \xi. \end{aligned}$$

The following remark describes the positivity of the Green’s function of the Lidstone interpolation.

### Remark 2

Clearly $G_{1}(x,s) \leq0$ and (26) yields $G_{n}(x,s)\geq0$ for even *n* and $G_{n}(x,s) \leq0$ for odd *n* for every $x,s\in[0,1]$.

The next section contains the main results of the paper.

## Main results

Throughout this section we use the following notations: $S_{1}(f,g,a,b)=f(g(a))-f(g(b))+\int_{a}^{b}f^{\prime}(t)g^{\prime}(t) \,dt$, $S_{2}(f,h,b)=\int_{0}^{b}f^{\prime}(t)h(t) \,dt-f ( \int_{0}^{b}h(t) \,dt ) $ and $S_{3}(f,h,k,b)=\int_{0}^{b}K(t)f^{\prime }(t)\times h(t) \,dt-\int_{0}^{b}k(x)f ( \int_{0}^{x}h(t) \,dt ) \,dx$. We start the section with the following theorem which enables us to obtain a generalization of (4).

### Theorem 2

*Let* $n\in\mathbb{N}$ *with* $n\geq2$ *and* $f:[a,b]\rightarrow\mathbb{R} $ *be* 2*n* *times differentiable function*. *Let* $g:[a,b]\rightarrow\mathbb{R}$ *be a non*-*decreasing function with* $g(a),g(b)\in{}[ a,b]$ *then*:

- *For* $j=1,2,4,5$, *we have*
  $$\begin{aligned} &S_{1}(f,g,a,b) \\ &\quad=\sum_{i=0}^{n-2} (b-a)^{2i} \biggl[ f^{(2i+2)} (a) \int _{a}^{b}S_{1} \bigl(G_{*,j}( \cdot,s),g,a,b \bigr)\Lambda_{i} \biggl(\frac{b-s}{b-a} \biggr) \,ds\\ &\qquad{}+ f^{(2i+2)}(b) \int_{a}^{b}S_{1} \bigl(G_{*,j}( \cdot,s),g,a,b \bigr) \Lambda_{i} \biggl(\frac {s-a}{b-a} \biggr) \,ds \biggr] \\ &\qquad{}+(b-a)^{2n-3} \int_{a}^{b}S_{1} \bigl(G_{*,j}( \cdot,s),g,a,b \bigr) \int_{a}^{b} G_{n-1} \biggl( \frac {s-a}{b-a},\frac{\xi-a}{b-a} \biggr)f^{(2n)}(\xi) \,d \xi\, ds. \end{aligned}$$
- *If* $f'(a)=0$ *then*
  $$\begin{aligned} &S_{1}(f,g,a,b) \\ &\quad=\sum_{i=0}^{n-2} (b-a)^{2i} \biggl[ f^{(2i+2)} (a) \int _{a}^{b}S_{1} \bigl(G_{*,3}( \cdot,s),g,a,b \bigr)\Lambda_{i} \biggl(\frac{b-s}{b-a} \biggr) \,ds\\ &\qquad{}+ f^{(2i+2)}(b) \int_{a}^{b}S_{1} \bigl(G_{*,3}( \cdot,s),g,a,b \bigr) \Lambda_{i} \biggl(\frac {s-a}{b-a} \biggr) \,ds \biggr] \\ &\qquad{}+ (b-a)^{2n-3} \int_{a}^{b}S_{1} \bigl(G_{*,3}( \cdot,s),g,a,b \bigr) \int_{a}^{b} G_{n-1} \biggl( \frac {s-a}{b-a},\frac{\xi-a}{b-a} \biggr)f^{(2n)}(\xi) \,d \xi\, ds, \end{aligned}$$

*where* $G_{*,j}(x,s)$ *is given by* (12)*–*(16).

### Proof

- We prove it for the case when $j=1$, the other cases $j=2,4,5$ are similar to this proof. By using (7) and (21) for *f* and $f^{\prime}$, respectively, we have
  $$\begin{aligned} S_{1}(f,g,a,b)={}&f \bigl(g(a) \bigr)-f \bigl(g(b) \bigr)+ \int_{a}^{b}f^{\prime}(t)g^{\prime}(t) \,dt\\ ={}& \frac{b-g(a)}{b-a}f(a)+\frac{g(a)-a}{b-a}f(b) \\ &{}+\int_{a}^{b}G_{*,1} \bigl(g(a),s \bigr)f^{\prime\prime}(s) \,ds-\frac{b-g(b)}{b-a }f(a)-\frac{g(b)-a}{b-a}f(b)\\ &{}- \int_{a}^{b}G_{*,1} \bigl(g(b),s \bigr)f^{\prime\prime}(s) \,ds \\ &{}+ \int_{a}^{b} \biggl[ \frac{f(b)-f(a )}{b-a}+ \int_{a}^{b}p_{1}(t,s)f^{\prime \prime}(s) \,ds \biggr] g^{\prime}(t) \,dt. \end{aligned}$$
  By simplifying and using Fubini’s theorem, we have
  $$\begin{aligned} &S_{1}(f,g,a,b)\\ &\quad=\frac{g(b)-g(a)}{b-a }f(a)-\frac{g(b)-g(a)}{b-a}f(b) \\ &\qquad{}+ \int_{a}^{b} \bigl[ G_{*,1} \bigl(g(a),s \bigr)-G_{*,1} \bigl(g(b),s \bigr) \bigr] f^{\prime \prime}(s) \,ds+\frac{f(b)-f(a)}{b-a} \bigl( g(b)-g(a) \bigr)\\ &\qquad{} + \int_{a}^{b} \int_{a}^{b}p_{1}(t,s)g^{\prime}(t)f^{\prime\prime}(s) \,dt\, ds \\ &\quad= \int_{a}^{b} S_{1} \bigl(G_{*,1}( \cdot,s),g,a,b \bigr)f^{\prime\prime}(s) \,ds. \end{aligned}$$
  Further, by substituting *n* with $n-1$ in (27) for $f^{\prime \prime}$, we get
  $$\begin{aligned} S_{1}(f,g,a,b)={}& \int_{a}^{b}S_{1} \bigl(G_{*,1}( \cdot,s),g,a,b \bigr) \Biggl( \sum_{i=0}^{n-2} (b-a)^{2i} f^{(2i+2)} (a) \Lambda_{i} \biggl( \frac{b-s}{b-a} \biggr)\\ &{}+ \sum_{i=0}^{n-2}(b-a)^{2i} f^{(2i+2)}(b) \Lambda_{i} \biggl(\frac{s-a}{b-a} \biggr) \\ &{}+(b-a)^{2n-3} \int_{a}^{b} G_{n-1} \biggl( \frac{s-a}{b-a},\frac{\xi-a}{b-a} \biggr) f^{(2n)}(\xi) \,d \xi \Biggr) \,ds, \end{aligned}$$
  which upon simplification gives the required identities.
- The proof is similar to part (a) except the use of the assumption $f'(a)=0$.

 □

The following theorem gives a generalized Steffensen’s inequality.

### Theorem 3

*Let* $n\in\mathbb{N}$ *with* $n\geq2$ *and let* $f:[a,b]\rightarrow\mathbb {R}$ *be* 2*n* *times differentiable and* $g:[a,b]\rightarrow\mathbb{R}$ *be a non*-*decreasing with* $g(a),g(b)\in{}[ a,b]$.

- *If* *f* *is* 2*n*-*convex and* *n* *is odd*, *then*
  $$\begin{aligned} S_{1}(f,g,a,b) \geq{}&\sum_{i=0}^{n-2} (b-a)^{2i} \biggl[ f^{(2i+2)} (a) \int _{a}^{b}S_{1} \bigl(G_{*,j}( \cdot,s),g,a,b \bigr)\Lambda_{i} \biggl(\frac{b-s}{b-a} \biggr) \,ds\\ &{}+ f^{(2i+2)}(b) \int_{a}^{b}S_{1} \bigl(G_{*,j}( \cdot,s),g,a,b \bigr) \Lambda_{i} \biggl(\frac {s-a}{b-a} \biggr) \,ds \biggr] \end{aligned}$$
  *for* $j=1,2,\ldots, 5$, *where* $f'(a)=0$ *for* $j=3$.
- *If* *f* *is* 2*n*-*convex and* *n* *is even*, *then the reverse of the inequality in part* (a) *holds*.
- *If* −*f* *is* 2*n*-*convex and* *n* *is odd*, *then the reverse of the inequality in part* (a) *holds*.
- *If* −*f* *is* 2*n*-*convex and* *n* *is even*, *then inequality in part* (a) *holds*.

### Proof

- For fix *s* and any $j\in\{1,2,3,4,5\}$, the function $G_{*,j}(\cdot,s)$ is convex and differentiable and since *g* is non-decreasing, therefore Corollary 1(a) gives $S_{1}(G_{*,j}(\cdot,s),g,a,b)\geq 0$. Moreover, 2*n*-convexity of *f* implies $f^{(2n)}(x)\geq0$ for $x\in {}[ a,b]$. Since $n-1$ is even, Remark 2 implies $G_{n-1}(\frac{s-a}{b-a},\frac{\xi-a}{b-a}) \geq0$. Thus, we have
  28 $$ (b-a)^{2n-3} \int_{a}^{b}S_{1} \bigl(G_{*,j}( \cdot,s),g,a,b \bigr) \int_{a}^{b} G_{n-1} \biggl( \frac {s-a}{b-a},\frac{\xi-a}{b-a} \biggr)f^{(2n)}(\xi) \,d \xi\, ds \geq0. $$
  Using this fact in Theorem 2 we get the desired inequality.
- The proof is similar to the part (a) except the fact that *f* is 2*n*-convex, therefore, $f^{(2n)}(x)\geq0$, $n-1$ odd implies $G_{n-1}(\frac{s-a}{b-a},\frac{\xi-a}{b-a}) \leq0$. Therefore, the reverse of (28) holds, which proves part (b).
- It follows from the facts that, under the assumptions, $f^{(2n)}(x)\leq0$ and $G_{n-1}(\frac{s-a}{b-a},\frac{\xi-a}{b-a}) \geq0$, which give the reverse of (28) and prove (c).
- It follows from the facts that, under the assumptions, $f^{(2n)}(x)\leq0$ and $G_{n-1}(\frac{s-a}{b-a},\frac{\xi-a}{b-a}) \leq0$, which yield (28) and complete the proof.

 □

In particular, the above theorem gives $S_{1}(f,g,a,b) \geq0$ and $S_{1}(f,g,a,b) \leq0$, which give (4) and its reverse. Consequently, Theorem 3 produces a generalization of Steffensen’s inequality and its reverse. Now, we prove the following theorem, which enables us to prove the generalization of (5).

### Theorem 4

*Let* $n\in\mathbb{N}$ *with* $n\geq2$ *and let* $f:[0,b]\rightarrow \mathbb{R}$ *be* 2*n* *times differentiable function with* $f(0)=0$. *If* $h:[0,b] \to[0,+\infty)$ *be an integrable function then*:

- $$\begin{aligned} S_{2}(f,h,b) ={}& \sum_{i=0}^{n-2} b^{2i} \biggl[ f^{(2i+2)} (0) \int _{0}^{b}S_{2} \bigl(G_{*,j}( \cdot,s),h,b \bigr)\Lambda_{i} \biggl(\frac{b-s}{b} \biggr)\,ds\\ &{}+ f^{(2i+2)}(b) \int_{0}^{b}S_{2} \bigl(G_{*,j}( \cdot,s),h,b \bigr) \Lambda_{i} \biggl(\frac{s}{b} \biggr) \,ds \biggr] \\ &{}+ b^{2n-3} \int_{0}^{b}S_{2} \bigl(G_{*,j}( \cdot,s),h,b \bigr) \biggl( \int_{0}^{b} G_{n-1} \biggl( \frac{s}{b},\frac{\xi}{b} \biggr)f^{(2n)}(\xi) \,d \xi \biggr) \,ds \end{aligned}$$
  *for* $j=1,2$.
- *If* $f'(0)=0$ *then*
  $$\begin{aligned} &S_{2}(f,h,b)+f(b) \\ &\quad =\sum_{i=0}^{n-2} b^{2i} \biggl[ f^{(2i+2)} (0) \int _{0}^{b}S_{2} \bigl(G_{*,3}( \cdot,s),h,b \bigr)\Lambda_{i} \biggl(\frac{b-s}{b} \biggr)\,ds\\ &\qquad{}+ f^{(2i+2)}(b) \int_{0}^{b}S_{2} \bigl(G_{*,3}( \cdot,s),h,b \bigr) \Lambda_{i} \biggl(\frac{s}{b} \biggr) \,ds \biggr] \\ &\qquad{}+ b^{2n-3} \int_{0}^{b}S_{2} \bigl(G_{*,3}( \cdot,s),h,b \bigr) \biggl( \int_{0}^{b} G_{n-1} \biggl( \frac{s}{b},\frac{\xi}{b} \biggr)f^{(2n)}(\xi) \,d \xi \biggr) \,ds. \end{aligned}$$
- $$\begin{aligned} &S_{2}(f,h,b)+f(b)-b f'(b) \\ &\quad=\sum_{i=0}^{n-2} b^{2i} \biggl[ f^{(2i+2)} (0) \int_{0}^{b}S_{2} \bigl(G_{*,4}( \cdot,s),h,b \bigr)\Lambda_{i} \biggl(\frac{b-s}{b} \biggr)\,ds\\ &\qquad{}+ f^{(2i+2)}(b) \int_{0}^{b}S_{2} \bigl(G_{*,4}( \cdot,s),h,b \bigr) \Lambda_{i} \biggl(\frac{s}{b} \biggr) \,ds \biggr] \\ &\qquad{}+ b^{2n-3} \int_{0}^{b}S_{2} \bigl(G_{*,4}( \cdot,s),h,b \bigr) \biggl( \int_{0}^{b} G_{n-1} \biggl( \frac{s}{b},\frac{\xi}{b} \biggr)f^{(2n)}(\xi) \,d \xi \biggr) \,ds. \end{aligned}$$
- *If* $f'(0)=0$ *then*
  $$\begin{aligned} &S_{2}(f,h,b)-b f'(b) \\ &\quad=\sum_{i=0}^{n-2} b^{2i} \biggl[ f^{(2i+2)} (0) \int_{0}^{b}S_{2} \bigl(G_{*,5}( \cdot,s),h,b \bigr)\Lambda_{i} \biggl(\frac{b-s}{b} \biggr)\,ds\\ &\qquad{}+ f^{(2i+2)}(b) \int_{0}^{b}S_{2} \bigl(G_{*,5}( \cdot,s),h,b \bigr) \Lambda_{i} \biggl(\frac{s}{b} \biggr) \,ds \biggr] \\ &\qquad{}+ b^{2n-3} \int_{0}^{b}S_{2} \bigl(G_{*,5}( \cdot,s),h,b \bigr) \biggl( \int_{0}^{b} G_{n-1} \biggl( \frac{s}{b},\frac{\xi}{b} \biggr)f^{(2n)}(\xi) \,d \xi \biggr) \,ds. \end{aligned}$$

### Proof

First, we prove for $j=1$, the proof of other cases are similar. By using (7) and (21) for *f* and $f^{\prime}$, respectively, and using the assumption that $f(0)=0$, we have

$$\begin{aligned} S_{2}(f,h,b)={}& \int_{0}^{b}f^{\prime}(t)h(t) \,dt-f \biggl( \int_{0}^{b}h(t) \,dt \biggr) \\ ={}&\int_{0}^{b}\frac{1}{b}f(b)h(t) \,dt+ \int_{0}^{b} \biggl[ \int_{0}^{b}{G_{*,1}}_{t}(t,s)f^{\prime\prime}(s) \,ds \biggr] h(t) \,dt-\frac{\int_{0}^{b}h(t) \,dt}{b}f(b)\\ &{}- \int_{0}^{b}G_{*,1} \biggl( \int_{0}^{b}h(t) \,dt,s \biggr) f^{\prime\prime}(s) \,ds \\ ={}& \int_{0}^{b}S_{2} \bigl(G_{*,1}( \cdot,s),h,b \bigr)f^{\prime\prime}(s) \,ds. \end{aligned}$$

Further, by substituting *n* with $n-1$ in (27) for $f^{\prime \prime}$ and simplifying we get the required identities. □

In the next theorem, we prove a generalization of (5) and its reverse.

### Theorem 5

*Let* $n\in\mathbb{N}$ *with* $n\geq2$ *and let* $f:[0,b]\rightarrow \mathbb{R}$ *be* 2*n* *times differentiable function with* $f(0)=0$ *and* *h* *be as in Corollary *2(a).

- *If* *f* *is* 2*n*-*convex and* *n* *is odd*, *then*:
  (i) *For* $j=1,2$, *we have*
    $$\begin{aligned} &S_{2}(f,h,b) \\ &\quad \geq\sum_{i=0}^{n-2} b^{2i} \biggl[ f^{(2i+2)} (0) \int _{0}^{b}S_{2} \bigl(G_{*,j}( \cdot,s),h,b \bigr)\Lambda_{i} \biggl(\frac{b-s}{b} \biggr)\,ds\\ &\qquad{}+ f^{(2i+2)}(b) \int_{0}^{b}S_{2} \bigl(G_{*,j}( \cdot,s),h,b \bigr) \Lambda_{i} \biggl(\frac{s}{b} \biggr) \,ds \biggr]. \end{aligned}$$
  (ii) *If* $f'(0)=0$ *then*
    $$\begin{aligned} &S_{2}(f,h,b)+f(b) \\ &\quad \geq\sum_{i=0}^{n-2} b^{2i} \biggl[ f^{(2i+2)} (0) \int_{0}^{b}S_{2} \bigl(G_{*,3}( \cdot,s),h,b \bigr)\Lambda_{i} \biggl(\frac{b-s}{b} \biggr)\,ds\\ &\qquad{}+ f^{(2i+2)}(b) \int_{0}^{b}S_{2} \bigl(G_{*,3}( \cdot,s),h,b \bigr) \Lambda_{i} \biggl(\frac{s}{b} \biggr) \,ds \biggr]. \end{aligned}$$
  (iii) $$\begin{aligned} &S_{2}(f,h,b)+f(b)-b f'(b) \\ &\quad \geq\sum_{i=0}^{n-2} b^{2i} \biggl[ f^{(2i+2)} (0) \int_{0}^{b}S_{2} \bigl(G_{*,4}( \cdot,s),h,b \bigr)\Lambda_{i} \biggl(\frac {b-s}{b} \biggr)\,ds\\ &\qquad{}+ f^{(2i+2)}(b) \int_{0}^{b}S_{2} \bigl(G_{*,4}( \cdot,s),h,b \bigr) \Lambda _{i} \biggl(\frac{s}{b} \biggr) \,ds \biggr]. \end{aligned}$$
  (iv) *If* $f'(0)=0$ *then*
    $$\begin{aligned} &S_{2}(f,h,b)-b f'(b) \\ &\quad\geq\sum_{i=0}^{n-2} b^{2i} \biggl[ f^{(2i+2)} (0) \int_{0}^{b}S_{2} \bigl(G_{*,5}( \cdot,s),h,b \bigr)\Lambda_{i} \biggl(\frac{b-s}{b} \biggr)\,ds\\ &\qquad{}+ f^{(2i+2)}(b) \int_{0}^{b}S_{2} \bigl(G_{*,5}( \cdot,s),h,b \bigr) \Lambda_{i} \biggl(\frac{s}{b} \biggr) \,ds \biggr]. \end{aligned}$$
- *If* *f* *is* 2*n*-*convex and* *n* *is even*, *then for each* *j* *the reverse of the inequality in part* (a) *holds*.
- *If* −*f* *is* 2*n*-*convex and* *n* *is odd*, *then for each* *j* *the reverse of the inequality in part* (a) *holds*.
- *If* -*f* *is* 2*n*-*convex and* *n* *is even*, *then for each* *j* *inequality in part* (a) *holds*.

### Proof

The proof can be obtained from Theorem 4 and Corollary 2(a) along the same lines as Theorem 3 has been proved by using Theorem 2 and Corollary 1(a). □

Now, we prove identities to obtain a generalization of (6).

### Theorem 6

*Let* $n\in\mathbb{N}$ *with* $n\geq2$ *and let* $f:[0,b]\rightarrow \mathbb{R}$ *be* 2*n* *times differentiable function with* $f(0)=0$ *and* *k* *and* *K* *be as in Corollary *3. *If* $h:[0,b] \to[0,+\infty)$ *is integrable then*:

- *For* $j=1,2$, *we have*
  $$\begin{aligned} &S_{3}(f,h,k,b) \\ &\quad=\sum_{i=0}^{n-2} b^{2i} \biggl[ f^{(2i+2)} (0) \int_{0}^{b} S_{3} \bigl(G_{*,j}( \cdot,s),h,k,b \bigr)\Lambda_{i} \biggl(\frac{b-s}{b} \biggr) \,ds\\ &\qquad{}+ f^{(2i+2)}(b) \int _{0}^{b} S_{3} \bigl(G_{*,j}( \cdot,s),h,k,b \bigr)\Lambda_{i} \biggl( \frac{s}{b} \biggr) \,ds \biggr] \\ &\qquad{}+b^{2n-3} \int_{0}^{b} S_{3} \bigl(G_{*,j}( \cdot,s),h,k,b \bigr) \biggl( \int_{0}^{b} G_{n-1} \biggl( \frac{s}{b},\frac{\xi}{b} \biggr)f^{(2n)}(\xi) \,d \xi \biggr) \,ds. \end{aligned}$$
- *If* $f'(0)=0$ *then*
  $$\begin{aligned} &S_{3}(f,h,k,b)+f(b) \int_{0}^{b}k(x) \,dx \\ &\quad =\sum_{i=0}^{n-2} b^{2i} \biggl[ f^{(2i+2)} (0) \int_{0}^{b} S_{3} \bigl(G_{*,3}( \cdot,s),h,k,b \bigr)\Lambda_{i} \biggl(\frac{b-s}{b} \biggr) \,ds\\ &\qquad{}+ f^{(2i+2)}(b) \int _{0}^{b} S_{3} \bigl(G_{*,3}( \cdot,s),h,k,b \bigr)\Lambda_{i} \biggl( \frac{s}{b} \biggr) \,ds \biggr] \\ &\qquad{}+b^{2n-3} \int_{0}^{b} S_{3} \bigl(G_{*,3}( \cdot,s),h,k,b \bigr) \biggl( \int_{0}^{b} G_{n-1} \biggl( \frac{s}{b},\frac{\xi}{b} \biggr)f^{(2n)}(\xi) \,d \xi \biggr) \,ds. \end{aligned}$$
- $$\begin{aligned} &S_{3}(f,h,k,b)+ \bigl(f(b)-b f'(b) \bigr) \int_{0}^{b}k(x) \,dx \\ &\quad=\sum_{i=0}^{n-2} b^{2i} \biggl[ f^{(2i+2)} (0) \int_{0}^{b} S_{3} \bigl(G_{*,4}( \cdot,s),h,k,b \bigr)\Lambda_{i} \biggl(\frac{b-s}{b} \biggr) \,ds\\ &\qquad{}+ f^{(2i+2)}(b) \int _{0}^{b} S_{3} \bigl(G_{*,4}( \cdot,s),h,k,b \bigr)\Lambda_{i} \biggl( \frac{s}{b} \biggr) \,ds \biggr] \\ &\qquad{}+b^{2n-3} \int_{0}^{b} S_{3} \bigl(G_{*,4}( \cdot,s),h,k,b \bigr) \biggl( \int_{0}^{b} G_{n-1} \biggl( \frac{s}{b},\frac{\xi}{b} \biggr)f^{(2n)}(\xi) \,d \xi \biggr) \,ds. \end{aligned}$$
- *If* $f'(0)=0$ *then*
  $$\begin{aligned} &S_{3}(f,h,k,b)-b f'(b) \int_{0}^{b}k(x) \,dx \\ &\quad=\sum_{i=0}^{n-2} b^{2i} \biggl[ f^{(2i+2)} (0) \int_{0}^{b} S_{3} \bigl(G_{*,5}( \cdot,s),h,k,b \bigr)\Lambda_{i} \biggl(\frac{b-s}{b} \biggr) \,ds\\ &\qquad{}+ f^{(2i+2)}(b) \int _{0}^{b} S_{3} \bigl(G_{*,5}( \cdot,s),h,k,b \bigr)\Lambda_{i} \biggl( \frac{s}{b} \biggr) \,ds \biggr] \\ &\qquad{}+b^{2n-3} \int_{0}^{b} S_{3} \bigl(G_{*,5}( \cdot,s),h,k,b \bigr) \biggl( \int_{0}^{b} G_{n-1} \biggl( \frac{s}{b},\frac{\xi}{b} \biggr)f^{(2n)}(\xi) \,d \xi \biggr) \,ds. \end{aligned}$$

### Proof

We prove the result for $j=1$. The proofs of the other parts are similar. By using (7) and (21) for *f* and $f^{\prime}$, respectively, we have

$$\begin{aligned} S_{3}(f,h,k,b)={}& \int_{0}^{b}K(t)f^{\prime}(t)h(t) \,dt- \int _{0}^{b}k(x)f \biggl( \int_{0}^{x}h(t) \,dt \biggr) \,dx\\ ={}& \int_{0}^{b}K(t)h(t) \biggl[ \frac{1}{b}f(b)+ \int _{0}^{b}{G_{*,1}}_{t}(t,s)f^{\prime \prime}(s) \,ds \biggr] \,dt\\ &{}- \int_{0}^{b}k(x) \biggl[ \frac{1}{b}f(b) \int_{0}^{x}h(t) \,dt + \int_{0}^{b}G_{*,1} \biggl( \int_{0}^{x}h(t) \,dt,s \biggr) f^{\prime\prime}(s) \,ds \biggr] \,dx \\ ={}&\frac{1}{b}f(b) \biggl[ \int_{0}^{b}K(t)h(t) \,dt- \int_{0}^{b}k(x)\int_{0}^{x}h(t) \,dt\, dx \biggr] \\ &{}+ \int_{0}^{b}K(t)h(t) \int_{0}^{b}{G_{*,1}}_{t}(t,s)f^{\prime\prime }(s) \,ds\, dt\\ &{}- \int_{0}^{b}k(x) \int_{0}^{b}G_{*,1} \biggl( \int_{0}^{x}h(t) \,dt,s \biggr) f^{\prime\prime}(s) \,ds\, dx. \end{aligned}$$

Since $\int_{0}^{b}k(x)\int_{0}^{x}h(t) \,dt\, dx=\int_{0}^{b}h(t) ( \int_{t}^{b}k(x) \,dx ) \,dt=\int_{0}^{b}K(t)h(t)\,dt$,

$$\begin{aligned} S_{3}(f,h,k,b) &= \int_{0}^{b} \biggl[ \int _{0}^{b}K(t)h(t){G_{*,1}}_{t}(t,s) \,dt-\int_{0}^{b}k(x)G_{*,1} \biggl( \int_{0}^{x}h(t) \,dt,s \biggr) \,dx \biggr] f^{\prime \prime}(s) \,ds\\ &= \int_{0}^{b}S_{3} \bigl(G_{*,1}( \cdot,s),h,k,b \bigr)f^{\prime\prime}(s) \,ds. \end{aligned}$$

The rest follows from (27). □

The following theorem gives a generalization of (6) and its reverse.

### Theorem 7

*Let* $n\in\mathbb{N}$ *with* $n\geq2$ *and let* $f:[0,b]\rightarrow\mathbb{R}$ *be* 2*n* *times differentiable function with* $f(0)=0$ *and* *k*, *K* *and* *h* *be as in Corollary *3(a).

- *If* *f* *is* 2*n*-*convex and* *n* *is odd*, *then*:
  (i) *For* $j=1,2$, *we have*
    $$\begin{aligned} &S_{3}(f,h,k,b) \\ &\quad\geq\sum_{i=0}^{n-2} b^{2i} \biggl[ f^{(2i+2)} (0) \int_{0}^{b} S_{3} \bigl(G_{*,j}( \cdot,s),h,k,b \bigr)\Lambda_{i} \biggl(\frac{b-s}{b} \biggr) \,ds\\ &\qquad{}+ f^{(2i+2)}(b) \int _{0}^{b} S_{3} \bigl(G_{*,j}( \cdot,s),h,k,b \bigr)\Lambda_{i} \biggl( \frac{s}{b} \biggr) \,ds \biggr]. \end{aligned}$$
  (ii) *If* $f'(0)=0$ *then*
    $$\begin{aligned} &S_{3}(f,h,k,b)+f(b) \int_{0}^{b}k(x) \,dx\\ &\quad \geq \sum_{i=0}^{n-2} b^{2i} \biggl[ f^{(2i+2)} (0) \int_{0}^{b} S_{3} \bigl(G_{*,3}( \cdot,s),h,k,b \bigr)\Lambda_{i} \biggl(\frac{b-s}{b} \biggr) \,ds\\ &\qquad{}+ f^{(2i+2)}(b) \int _{0}^{b} S_{3} \bigl(G_{*,3}( \cdot,s),h,k,b \bigr)\Lambda_{i} \biggl( \frac{s}{b} \biggr) \,ds \biggr]. \end{aligned}$$
  (iii) $$\begin{aligned} &S_{3}(f,h,k,b)+ \bigl(f(b)-b f'(b) \bigr) \int_{0}^{b}k(x) \,dx \\ &\quad \geq \sum_{i=0}^{n-2} b^{2i} \biggl[ f^{(2i+2)} (0) \int_{0}^{b} S_{3} \bigl(G_{*,4}( \cdot,s),h,k,b \bigr)\Lambda_{i} \biggl(\frac{b-s}{b} \biggr) \,ds\\ &\qquad{}+ f^{(2i+2)}(b) \int _{0}^{b} S_{3} \bigl(G_{*,4}( \cdot,s),h,k,b \bigr)\Lambda_{i} \biggl( \frac{s}{b} \biggr) \,ds \biggr]. \end{aligned}$$
  (iv) *If* $f'(0)=0$ *then*
    $$\begin{aligned} &S_{3}(f,h,k,b)-b f'(b) \int_{0}^{b}k(x) \,dx\\ &\quad \geq \sum_{i=0}^{n-2} b^{2i} \biggl[ f^{(2i+2)} (0) \int_{0}^{b} S_{3} \bigl(G_{*,5}( \cdot,s),h,k,b \bigr)\Lambda_{i} \biggl(\frac{b-s}{b} \biggr) \,ds\\ &\qquad{}+ f^{(2i+2)}(b) \int _{0}^{b} S_{3} \bigl(G_{*,5}( \cdot,s),h,k,b \bigr)\Lambda_{i} \biggl( \frac{s}{b} \biggr) \,ds \biggr]. \end{aligned}$$
- *If* *f* *is* 2*n*-*convex and* *n* *is even*, *then for each* *j* *the reverse of the inequality in part* (a) *holds*.
- *If* −*f* *is* 2*n*-*convex and* *n* *is odd*, *then for each* *j* *the reverse of the inequality in part* (a) *holds*.
- *If* -*f* *is* 2*n*-*convex and* *n* *is even*, *then for each* *j* *inequality in part* (a) *holds*.

### Proof

The proof follows from Theorem 6 and Corollary 3(a) in a similar way to the proof of Theorem 3 by using Theorem 2 and Corollary 1(a). □

## Application to $(2n+1)$-convex function at a point

In the present section we prove results related to the following $(n+1)$-convex function at a point introduced in [9]. Let $I\subseteq\mathbb{R}$ be an interval, $c \in I^{0}$ and $n\in\mathbb{N}$. A function $f: I \rightarrow\mathbb{R}$ is said to be $(n + 1)$-convex at point *c* if there exists a constant $K_{c}$ such that the function $F(x) = f(x)- K_{c} \frac{x^{n}}{n!}$ is *n*-concave on $I \cap(-\infty, c]$ and *n*-convex on $I \cap[c,\infty)$. A function *f* is said to be $(n + 1)$-concave at point *c* if the function −*f* is $(n + 1)$-convex at point *c*. Pečarić, Praljak and Witkowski in [9] studied necessary and sufficient conditions on two linear functionals $\Omega_{1}: C([\delta_{1}, c])\rightarrow\mathbb{R}$ and $\Gamma_{1}: C([c, \delta _{2}]\rightarrow\mathbb{R}$ so that the inequality $\Omega_{1}(f) \leq \Gamma_{1} (f)$ holds for every function *f* that is $(n+1)$-convex at point *c*. In this section, we define linear functionals and obtain such inequalities for defined functionals. Let $n\in \mathbb{N}$ be odd with $n>2$, $f: [a,b] \rightarrow\mathbb{R}$ be 2*n* times differentiable function, $a_{1}, a_{2} \in[a,b]$ and $c \in(a,b)$, where $a_{1} < c < a_{2}$. Let $g_{1}:[a_{1},c]\rightarrow\mathbb{R}$ and $g_{2}:[c,a_{2}]\rightarrow\mathbb{R}$ be non-decreasing with $g_{i}(x) \leq x$ for $i=1,2$. For $j=1,2, \dots, 5$, we define

29 $$\begin{aligned} \Omega_{1,j}(f)={}&S_{1}(f,g_{1},a_{1},c) \\ &{}- \sum_{i=0}^{n-2} (c-a_{1})^{2i} \biggl[ f^{(2i+2)} (a_{1}) \int_{a_{1}}^{c}S_{1} \bigl(G_{*,j}( \cdot,s),g_{1},a_{1},c \bigr)\Lambda _{i} \biggl( \frac{c-s}{c-a_{1}} \biggr)\,ds \\ &{}+ f^{(2i+2)}(c) \int _{a_{1}}^{c}S_{1} \bigl(G_{*,j}( \cdot,s),g_{1},a_{1},c \bigr) \Lambda_{i} \biggl( \frac{s-a_{1}}{c-a_{1}} \biggr) \,ds \biggr] \end{aligned}$$

and

30 $$\begin{aligned} \Gamma_{1,j}(f)={}&S_{1}(f,g_{2},c,a_{2}) \\ &{}- \sum_{i=0}^{n-2} (a_{2}-c)^{2i} \biggl[ f^{(2i+2)} (c) \int_{c}^{a_{2}}S_{1} \bigl(G_{*,j}( \cdot,s),g_{2},c,a_{2} \bigr)\Lambda_{i} \biggl( \frac {a_{2}-s}{a_{2}-c} \biggr)\,ds \\ &{}+ f^{(2i+2)}(a_{2}) \int _{c}^{a_{2}}S_{1} \bigl(G_{*,j}( \cdot,s),g_{2},c,a_{2} \bigr) \Lambda_{i} \biggl( \frac{s-c}{a_{2}-c} \biggr) \,ds \biggr]. \end{aligned}$$

Similarly let $c \in(0,b)$, $b_{1} \in(0,b]$ where $c < b_{1}$ and let $h_{1}:[0,c]\rightarrow[0,+\infty)$ and $h_{2}:[c,b_{1}]\rightarrow[0,+\infty)$ be as defined in Corollary 2(a) (w.l.o.g. we may assume $h_{2}$ on $[0,b_{1}]$ by taking $h_{2}(t)=0$ when $t\in[0,c]$). We define the following pair of functionals:

- $$\begin{aligned} \Omega_{2,j}(f)={}& S_{2}(f,h_{1},c)- \sum _{i=0}^{n-2} c^{2i} \biggl[ f^{(2i+2)} (0) \int_{0}^{c}S_{2} \bigl(G_{*,j}( \cdot,s),h_{1},c \bigr)\Lambda_{i} \biggl(\frac {c-s}{c} \biggr)\,ds \\ &{}+ f^{(2i+2)}(c) \int_{0}^{c}S_{2} \bigl(G_{*,j}( \cdot,s),h_{1},c \bigr) \Lambda_{i} \biggl( \frac{s}{c} \biggr) \,ds \biggr] \end{aligned}$$
  and
  $$\begin{aligned} \Gamma_{2,j}(f)={}& S_{2}(f,h_{2},b_{1})\\ &{}- \sum_{i=0}^{n-2} b_{1}^{2i} \biggl[ f^{(2i+2)} (0) \int_{c}^{b_{1}}S_{2} \bigl(G_{*,j}( \cdot,s),h_{2},b_{1} \bigr)\Lambda_{i} \biggl( \frac {b_{1}-s}{b_{1}} \biggr)\,ds \\ &{}+ f^{(2i+2)}(b_{1}) \int _{c}^{b_{1}}S_{2} \bigl(G_{*,j}( \cdot,s),h_{2},b_{1} \bigr) \Lambda_{i} \biggl( \frac{s}{b_{1}} \biggr) \,ds \biggr], \end{aligned}$$
  where $j=1,2$.
- $$\begin{aligned} \Omega_{2,3}(f)={}& S_{2}(f,h_{1},c)+f(c)\\ &{}- \sum _{i=0}^{n-2} c^{2i} \biggl[ f^{(2i+2)} (0) \int_{0}^{c}S_{2} \bigl(G_{*,3}( \cdot,s),h_{1},c \bigr)\Lambda_{i} \biggl(\frac {c-s}{c} \biggr)\,ds \\ &{}+ f^{(2i+2)}(c) \int_{0}^{c}S_{2} \bigl(G_{*,3}( \cdot,s),h_{1},c \bigr) \Lambda_{i} \biggl( \frac{s}{c} \biggr) \,ds \biggr] \end{aligned}$$
  and
  $$\begin{aligned} \Gamma_{2,3}(f)={}& S_{2}(f,h_{2},b_{1})+f(b_{1})\\ &{}- \sum_{i=0}^{n-2} b_{1}^{2i} \biggl[ f^{(2i+2)} (0) \int_{c}^{b_{1}}S_{2} \bigl(G_{*,3}( \cdot,s),h_{2},b_{1} \bigr)\Lambda_{i} \biggl( \frac {b_{1}-s}{b_{1}} \biggr)\,ds \\ &{}+ f^{(2i+2)}(b_{1}) \int _{c}^{b_{1}}S_{2} \bigl(G_{*,3}( \cdot,s),h_{2},b_{1} \bigr) \Lambda_{i} \biggl( \frac{s}{b_{1}} \biggr) \,ds \biggr]. \end{aligned}$$
- $$\begin{aligned} \Omega_{2,4}(f)= {}&S_{2}(f,h_{1},c)+f(c)-c f'(c)\\ &{}- \sum_{i=0}^{n-2} c^{2i} \biggl[ f^{(2i+2)} (0) \int_{0}^{c}S_{2} \bigl(G_{*,4}( \cdot,s),h_{1},c \bigr)\Lambda_{i} \biggl(\frac {c-s}{c} \biggr)\,ds \\ &{}+ f^{(2i+2)}(c) \int_{0}^{c}S_{2} \bigl(G_{*,4}( \cdot,s),h_{1},c \bigr) \Lambda_{i} \biggl( \frac{s}{c} \biggr) \,ds \biggr] \end{aligned}$$
  and
  $$\begin{aligned} \Gamma_{2,4}(f)={}& S_{2}(f,h_{2},b_{1})+f(b_{1})-b_{1} f'(b_{1})\\ &{}- \sum_{i=0}^{n-2} b_{1}^{2i} \biggl[ f^{(2i+2)} (0) \int _{c}^{b_{1}}S_{2} \bigl(G_{*,4}( \cdot,s),h_{2},b_{1} \bigr)\Lambda_{i} \biggl( \frac{b_{1}-s}{b_{1}} \biggr)\,ds \\ &{}+ f^{(2i+2)}(b_{1}) \int_{c}^{b_{1}}S_{2} \bigl(G_{*,4}( \cdot,s),h_{2},b_{1} \bigr) \Lambda_{i} \biggl( \frac {s}{b_{1}} \biggr) \,ds \biggr]. \end{aligned}$$
- $$\begin{aligned} \Omega_{2,5}(f)={}& S_{2}(f,h_{1},c)-c f'(c)\\ &{}- \sum_{i=0}^{n-2} c^{2i} \biggl[ f^{(2i+2)} (0) \int_{0}^{c}S_{2} \bigl(G_{*,5}( \cdot,s),h_{1},c \bigr)\Lambda_{i} \biggl(\frac {c-s}{c} \biggr)\,ds \\ &{}+ f^{(2i+2)}(c) \int_{0}^{c}S_{2} \bigl(G_{*,5}( \cdot,s),h_{1},c \bigr) \Lambda_{i} \biggl( \frac{s}{c} \biggr) \,ds \biggr] \end{aligned}$$
  and
  $$\begin{aligned} \Gamma_{2,5}(f)= {}&S_{2}(f,h_{2},b_{1})-b_{1} f'(b_{1})\\ &{}- \sum_{i=0}^{n-2} b_{1}^{2i} \biggl[ f^{(2i+2)} (0) \int_{c}^{b_{1}}S_{2} \bigl(G_{*,5}( \cdot,s),h_{2},b_{1} \bigr)\Lambda _{i} \biggl( \frac{b_{1}-s}{b_{1}} \biggr)\,ds \\ &{}+ f^{(2i+2)}(b_{1}) \int _{c}^{b_{1}}S_{2} \bigl(G_{*,5}( \cdot,s),h_{2},b_{1} \bigr) \Lambda_{i} \biggl( \frac{s}{b_{1}} \biggr) \,ds \biggr]. \end{aligned}$$

Lastly, we define:

- $$\begin{aligned} \Omega_{3,j}(f)={}&S_{3}(f,h_{1},k,c)- \sum _{i=0}^{n-2} c^{2i} \biggl[ f^{(2i+2)} (0) \int_{0}^{c} S_{3} \bigl(G_{*,j}( \cdot,s),h_{1},k,c \bigr)\Lambda_{i} \biggl( \frac{c-s}{c} \biggr) \,ds \\ &{}+ f^{(2i+2)}(c) \int_{0}^{c} S_{3} \bigl(G_{*,j}( \cdot,s),h_{1},k,c \bigr)\Lambda_{i} \biggl( \frac{s}{c} \biggr) \,ds \biggr] \end{aligned}$$
  and
  $$\begin{aligned} \Gamma_{3,j}(f)={}&S_{3}(f,h_{2},k,b_{1})\\ &{}- \sum_{i=0}^{n-2} b_{1}^{2i} \biggl[ f^{(2i+2)} (0) \int_{c}^{b_{1}} S_{3} \bigl(G_{*,j}( \cdot,s),h_{2},k,b_{1} \bigr)\Lambda_{i} \biggl( \frac {b_{1}-s}{b_{1}} \biggr) \,ds \\ &{}+ f^{(2i+2)}(b_{1}) \int_{c}^{b_{1}} S_{3} \bigl(G_{*,j}( \cdot,s),h_{2},k,b_{1} \bigr)\Lambda_{i} \biggl( \frac{s}{b_{1}} \biggr) \,ds \biggr], \end{aligned}$$
  where $j=1,2$.
- $$\begin{aligned} \Omega_{3,3}(f)={}& S_{3}(f,h_{1},k,c)+f(c) \int_{0}^{c} k(x) \,dx\\ &{}- \sum _{i=0}^{n-2} c^{2i} \biggl[ f^{(2i+2)} (0) \int_{0}^{c} S_{3} \bigl(G_{*,3}( \cdot,s),h_{1},k,c \bigr)\Lambda_{i} \biggl( \frac{c-s}{c} \biggr) \,ds \\ &{}+ f^{(2i+2)}(c) \int_{0}^{c} S_{3} \bigl(G_{*,3}( \cdot,s),h_{1},k,c \bigr)\Lambda_{i} \biggl( \frac{s}{c} \biggr) \,ds \biggr] \end{aligned}$$
  and
  $$\begin{aligned} \Gamma_{3,3}(f)={}& S_{3}(f,h_{2},k,b_{1})+f(b_{1}) \int_{c}^{b_{1}} k(x) \,dx\\ &{}-\sum _{i=0}^{n-2} b_{1}^{2i} \biggl[ f^{(2i+2)} (0) \int_{c}^{b_{1}} S_{3} \bigl(G_{*,3}( \cdot,s),h_{2},k,b_{1} \bigr)\Lambda_{i} \biggl( \frac{b_{1}-s}{b_{1}} \biggr) \,ds \\ &{}+ f^{(2i+2)}(b_{1}) \int_{c}^{b_{1}} S_{3} \bigl(G_{*,3}( \cdot,s),h_{2},k,b_{1} \bigr)\Lambda _{i} \biggl( \frac{s}{b_{1}} \biggr) \,ds \biggr]. \end{aligned}$$
- $$\begin{aligned} \Omega_{3,4}(f={}& S_{3}(f,h_{1},k,c)+ \bigl(f(c)-c f'(c) \bigr) \int_{0}^{c} k(x) \,dx\\ &{}- \sum _{i=0}^{n-2} c^{2i} \biggl[ f^{(2i+2)} (0) \int_{0}^{c} S_{3} \bigl(G_{*,4}( \cdot,s),h_{1},k,c \bigr)\Lambda_{i} \biggl( \frac{c-s}{c} \biggr) \,ds \\ &{}+ f^{(2i+2)}(c) \int_{0}^{c} S_{3} \bigl(G_{*,4}( \cdot,s),h_{1},k,c \bigr)\Lambda_{i} \biggl( \frac{s}{c} \biggr) \,ds \biggr] \end{aligned}$$
  and
  $$\begin{aligned} \Gamma_{3,4}(f) ={}& S_{3}(f,h_{2},k,b_{1})+ \bigl(f(b_{1})-b_{1} f'(b_{1}) \bigr) \int_{c}^{b_{1}} k(x) \,dx\\ &{}-\sum _{i=0}^{n-2} b_{1}^{2i} \biggl[ f^{(2i+2)} (0) \int_{c}^{b_{1}} S_{3} \bigl(G_{*,4}( \cdot,s),h_{2},k,b_{1} \bigr)\Lambda_{i} \biggl( \frac{b_{1}-s}{b_{1}} \biggr) \,ds \\ &{}+ f^{(2i+2)}(b_{1}) \int_{c}^{b_{1}} S_{3} \bigl(G_{*,4}( \cdot,s),h_{2},k,b_{1} \bigr)\Lambda _{i} \biggl( \frac{s}{b_{1}} \biggr) \,ds \biggr]. \end{aligned}$$
- $$\begin{aligned} \Omega_{3,5}(f)={}&S_{3}(f,h_{1},k,c)-c f'(c) \int_{0}^{c} k(x) \,dx\\ &{}- \sum _{i=0}^{n-2} c^{2i} \biggl[ f^{(2i+2)} (0) \int_{0}^{c} S_{3} \bigl(G_{*,5}( \cdot,s),h_{1},k,c \bigr)\Lambda_{i} \biggl( \frac{c-s}{c} \biggr) \,ds \\ &{}+ f^{(2i+2)}(c) \int_{0}^{c} S_{3} \bigl(G_{*,5}( \cdot,s),h_{1},k,c \bigr)\Lambda_{i} \biggl( \frac{s}{c} \biggr) \,ds \biggr] \end{aligned}$$
  and
  $$\begin{aligned} \Gamma_{3,5}(f)={}& S_{3}(f,h_{2},k,b_{1})-b_{1} f'(b_{1}) \int_{c}^{b_{1}} k(x) \,dx\\ &{}-\sum _{i=0}^{n-2} b_{1}^{2i} \biggl[ f^{(2i+2)} (0) \int_{c}^{b_{1}} S_{3} \bigl(G_{*,5}( \cdot,s),h_{2},k,b_{1} \bigr)\Lambda_{i} \biggl( \frac{b_{1}-s}{b_{1}} \biggr) \,ds \\ &{}+ f^{(2i+2)}(b_{1}) \int_{c}^{b_{1}} S_{3} \bigl(G_{*,5}( \cdot,s),h_{2},k,b_{1} \bigr)\Lambda _{i} \biggl( \frac{s}{b_{1}} \biggr) \,ds \biggr], \end{aligned}$$

where *k* is as defined in Corollary 3. If *f* is 2*n*-convex then Theorem 3(a), Theorem 5(a) and Theorem 7(a) implies $\Gamma_{1,j}(f) \geq0$, $\Gamma_{2,j}(f) \geq0$ and $\Gamma_{3,j}(f) \geq0$ for $j=1,2,\dots, 5$ (and $f'(0)=0$ for $j=3$), respectively. Moreover, if −*f* is 2*n*-convex then Theorem 3(c), Theorem 5(c) and Theorem 7(c) imply $\Omega _{1,j}(f) \leq0$, $\Omega_{2,j}(f) \leq0$ and $\Omega_{3,j}(f) \leq 0$ for $j=1,2,\dots, 5$ (and $f'(0)=0$ for $j=3$), respectively.

### Theorem 8

*Let* $n\in\mathbb{N}$ *with* $n> 2$ *be odd and let* $f:[a,b]\rightarrow\mathbb{R}$ *be* $(2n+1)$-*convex at a point* *c* *in* $(a,b)$. *Let* $g_{1}:[a_{1},c]\rightarrow\mathbb{R}$ *and* $g_{2}:[c,a_{2}]\rightarrow\mathbb{R}$, *where* $a_{1}< c < a_{2}$, *be non*-*decreasing and differentiable functions*. *If* $\Omega_{1,j}(\phi_{0})= \Gamma_{1,j}(\phi_{0})$, *for all* $j=1,2, \ldots, 5$ *and* $f'(a)=0$ *for* $j=3$, *where* $\phi_{0}(x)=x^{2n}$ *then*

$$ \Omega_{1,j}(f)\leq\Gamma_{1,j}(f), $$

*for* $j=1,2, \dots, 5$.

### Proof

Since *f* is $(2n+1)$-convex at *c*, there exists a $K_{c}$ such that $F(x)=f(x)-\frac{K_{c}x^{2n}}{(2n)!}$ is 2*n*-concave (or −*F* is 2*n*-convex) on $[a_{1},c]$ and 2*n*-convex on $[c,a_{2}]$. Therefore, for each $j=1,2, \dots, 5$, we have $0 \geq\Omega_{1,j}(F)= \Omega_{1,j}(f)-\frac{K_{c}}{(2n)!}\Omega_{1,j}(\phi_{0})$. Moreover, since *F* is 2*n*-convex on $[c,a_{2}]$, $0 \leq\Gamma_{1,j}(F)= \Gamma_{1,j}(f)-\frac{K_{c}}{(2n)!}\Gamma _{1,j}(\phi_{0})$. Since $\Omega_{1,j}(\phi_{0})= \Gamma_{1,j}(\phi_{0})$, $\Omega_{1,j}(f)\leq\Gamma_{1,j}(f)$, which completes the proof. □

### Theorem 9

*Let* $n\in\mathbb{N}$ *with* $n>2$ *be odd*, *let* $h_{1}:[0,c] \rightarrow[0,+\infty)$ *and* $h_{2}:[c,b_{1}] \rightarrow[0,+\infty)$ *be as defined in Corollary *2(a) *and* $k:[0,b] \rightarrow[0,+\infty)$ *be as in Corollary *3. *If* $f:[0,b]\rightarrow\mathbb{R}$ *is* $(2n+1)$-*convex at a point* *c* *in* $(0,b)$ *then*:

-
  (i) *If*
    $$\begin{aligned} &S_{2}(\phi_{0}, h_{1},c)-\sum _{i=0}^{n-2} c^{2i} \phi_{0}^{(2i+2)}(c) \int _{0}^{c} S_{2} \bigl(G_{*,j}( \cdot,s),h_{1},c \bigr) \Lambda_{i} \biggl( \frac{s}{c} \biggr) \,ds\\ &\quad= S_{2}(\phi_{0}, h_{2},b_{1})\\ &\qquad{}-\sum _{i=0}^{n-2} b_{1}^{2i} \phi_{0}^{(2i+2)}(b_{1}) \int _{c}^{b_{1}}S_{2} \bigl(G_{*,j}( \cdot,s),h_{2},b_{1} \bigr) \Lambda_{i} \biggl( \frac{s}{b_{1}} \biggr) \,ds \end{aligned}$$
    *then* $\Omega_{2,j}(f) \leq\Gamma_{2,j}(f)$ *for* $j=1,2$, *where* $\phi _{0}(x)=x^{2n}$.
  (ii) *If* $f'(0)=0$ *and*
    $$\begin{aligned} &S_{2}(\phi_{0}, h_{1},c)+c^{2n}\\ &\qquad{}-\sum _{i=0}^{n-2} c^{2i} \phi _{0}^{(2i+2)}(c) \int_{0}^{c} S_{2} \bigl(G_{*,3}( \cdot,s),h_{1},c \bigr) \Lambda_{i} \biggl( \frac {s}{c} \biggr) \,ds\\ &\quad= S_{2}(\phi_{0}, h_{2},b_{1})+b_{1}^{2n}\\ &\qquad{}- \sum_{i=0}^{n-2} b_{1}^{2i} \phi _{0}^{(2i+2)}(b_{1}) \int_{c}^{b_{1}}S_{2} \bigl(G_{*,3}( \cdot,s),h_{2},b_{1} \bigr) \Lambda_{i} \biggl( \frac {s}{b_{1}} \biggr) \,ds \end{aligned}$$
    *then* $\Omega_{2,3}(f) \leq\Gamma_{2,3}(f)$.
  (iii) *If*
    $$\begin{aligned} &S_{2}(\phi_{0}, h_{1},c)+(1-2n)c^{2n}\\ &\qquad{}- \sum_{i=0}^{n-2} c^{2i} \phi _{0}^{(2i+2)}(c) \int_{0}^{c} S_{2} \bigl(G_{*,4}( \cdot,s),h_{1},c \bigr) \Lambda_{i} \biggl( \frac {s}{c} \biggr) \,ds\\ &\quad= S_{2}(\phi_{0}, h_{2},b_{1})+(1-2n)b_{1}^{2n}\\ &\qquad{}- \sum_{i=0}^{n-2} b_{1}^{2i} \phi _{0}^{(2i+2)}(b_{1}) \int_{c}^{b_{1}}S_{2} \bigl(G_{*,4}( \cdot,s),h_{2},b_{1} \bigr) \Lambda_{i} \biggl( \frac {s}{b_{1}} \biggr) \,ds \end{aligned}$$
    *then* $\Omega_{2,4}(f) \leq\Gamma_{2,4}(f)$.
  (iv) *If*
    $$\begin{aligned} &S_{2}(\phi_{0}, h_{1},c)-2nc^{2n}-\sum _{i=0}^{n-2} c^{2i} \phi _{0}^{(2i+2)}(c) \int_{0}^{c} S_{2} \bigl(G_{*,5}( \cdot,s),h_{1},c \bigr) \Lambda_{i} \biggl( \frac {s}{c} \biggr) \,ds\\ &\quad = S_{2}(\phi_{0}, h_{2},b_{1})-2n b_{1}^{2n}\\ &\qquad{}- \sum_{i=0}^{n-2} b_{1}^{2i} \phi _{0}^{(2i+2)}(b_{1}) \int_{c}^{b_{1}}S_{2} \bigl(G_{*,5}( \cdot,s),h_{2},b_{1} \bigr) \Lambda_{i} \biggl( \frac {s}{b_{1}} \biggr) \,ds \end{aligned}$$
    *then* $\Omega_{2,5}(f) \leq\Gamma_{2,5}(f)$.
-
  (i) *If*
    $$\begin{aligned} &S_{3}(\phi_{0}, h_{1},k,c)-\sum _{i=0}^{n-2} c^{2i}\phi_{0}^{(2i+2)}(c) \int _{0}^{c} S_{3} \bigl(G_{*,j}( \cdot,s),h_{1},k,c \bigr)\Lambda_{i} \biggl(\frac{s}{c} \biggr) \,ds\\ &\quad= S_{3}(\phi_{0}, h_{2},k,b_{1})\\ &\qquad{}-\sum _{i=0}^{n-2} b_{1}^{2i} \phi_{0}^{(2i+2)}(b_{1}) \int_{c}^{b_{1}} S_{3} \bigl(G_{*,j}( \cdot,s),h_{2},k,b_{1} \bigr)\Lambda_{i} \biggl( \frac{s}{b_{1}} \biggr) \,ds \end{aligned}$$
    *then* $\Omega_{3,j}(f) \leq\Gamma_{3,j}(f)$ *for* $j=1,2$.
  (ii) *If* $f'(0)=0$ *and*
    $$\begin{aligned} &S_{3}(\phi_{0}, h_{1},k,c)+{c}^{2n} \int_{0}^{c} k(x) \,dx\\ &\qquad{}-\sum _{i=0}^{n-2} c^{2i}\phi_{0}^{(2i+2)}(c) \int_{0}^{c} S_{3} \bigl(G_{*,3}( \cdot,s),h_{1},k,c \bigr)\Lambda _{i} \biggl( \frac{s}{c} \biggr) \,ds\\ &\quad= S_{3}(\phi_{0}, h_{2},k,b_{1})+b_{1}^{2n} \int_{c}^{b_{1}} k(x) \,dx\\ &\qquad{}-\sum _{i=0}^{n-2} b_{1}^{2i} \phi_{0}^{(2i+2)}(b_{1}) \int_{c}^{b_{1}} S_{3} \bigl(G_{*,3}( \cdot,s),h_{2},k,b_{1} \bigr)\Lambda_{i} \biggl( \frac{s}{b_{1}} \biggr) \,ds \end{aligned}$$
    *then* $\Omega_{3,3}(f) \leq\Gamma_{3,3}(f)$.
  (iii) *If*
    $$\begin{aligned} &S_{3}(\phi_{0}, h_{1},k,c)+(1-2n){c}^{2n} \int_{0}^{c} k(x) \,dx\\ &\qquad{}-\sum _{i=0}^{n-2} c^{2i}\phi_{0}^{(2i+2)}(c) \int_{0}^{c} S_{3} \bigl(G_{*,4}( \cdot,s),h_{1},k,c \bigr)\Lambda_{i} \biggl( \frac{s}{c} \biggr) \,ds\\ &\quad= S_{3}(\phi_{0}, h_{2},k,b_{1})+(1-2n)b_{1}^{2n} \int_{c}^{b_{1}} k(x) \,dx\\ &\qquad{}-\sum _{i=0}^{n-2} b_{1}^{2i} \phi_{0}^{(2i+2)}(b_{1}) \int_{c}^{b_{1}} S_{3} \bigl(G_{*,4}( \cdot,s),h_{2},k,b_{1} \bigr)\Lambda_{i} \biggl( \frac{s}{b_{1}} \biggr) \,ds \end{aligned}$$
    *then* $\Omega_{3,4}(f) \leq\Gamma_{3,4}(f)$.
  (iv) *If*
    $$\begin{aligned} &S_{3}(\phi_{0}, h_{1},k,c)-2n{c}^{2n} \int_{0}^{c} k(x) \,dx\\ &\qquad{}-\sum _{i=0}^{n-2} c^{2i}\phi_{0}^{(2i+2)}(c) \int_{0}^{c} S_{3} \bigl(G_{*,5}( \cdot,s),h_{1},k,c \bigr)\Lambda _{i} \biggl( \frac{s}{c} \biggr) \,ds\\ &\quad = S_{3}(\phi_{0}, h_{2},k,b_{1})-2n b_{1}^{2n} \int_{c}^{b_{1}} k(x) \,dx\\ &\qquad{}-\sum _{i=0}^{n-2} b_{1}^{2i} \phi_{0}^{(2i+2)}(b_{1}) \int_{c}^{b_{1}} S_{3} \bigl(G_{*,5}( \cdot,s),h_{2},k,b_{1} \bigr)\Lambda_{i} \biggl( \frac{s}{b_{1}} \biggr) \,ds \end{aligned}$$
    *then* $\Omega_{3,5}(f) \leq\Gamma_{3,5}(f)$.

### Proof

- Since *f* is $(2n+1)$-convex at *c*, there exists a $K_{c}$ such that $F(x)=f(x)-\frac{K_{c}x^{2n}}{(2n)!}$ is 2*n*-concave (or −*F* is 2*n*-convex) on $[0,c]$ and 2*n*-convex on $[c,b_{1}]$. Therefore,
  $$\begin{aligned} 0 \geq{}&\Omega_{2,j}(F)\\ ={}& \Omega_{2,j}(f)\\ &{}-\frac{K_{c}}{(2n)!} \Biggl( \sum_{i=0}^{n-2} c^{2i} \phi_{0}^{(2i+2)}(c) \int_{0}^{c} S_{2} \bigl(G_{*,j}( \cdot,s),h_{1},c \bigr) \Lambda_{i} \biggl( \frac{s}{c} \biggr) \,ds-S_{2}(\phi_{0}, h_{1},c) \Biggr). \end{aligned}$$
  On the other hand, since *F* is 2*n*-convex on $[c,b_{1}]$,
  $$\begin{aligned} 0 \leq{}&\Gamma_{2,j}(F)\\ ={}& \Gamma_{2,j}(f)\\ &{}-\frac{K_{c}}{(2n)!} \Biggl(\sum_{i=0}^{n-2} b_{1}^{2i} \phi_{0}^{(2i+2)}(b_{1}) \int _{c}^{b_{1}}S_{2} \bigl(G_{*,j}( \cdot,s),h_{2},b_{1} \bigr) \Lambda_{i} \biggl( \frac{s}{b_{1}} \biggr) \,ds \\ &{}-S(\phi _{0}, h_{2},b_{1}) \Biggr). \end{aligned}$$
  So under the given assumption, we have $\Omega_{2,j}(f)\leq\Gamma _{2,j}(f)$ for $j=1,2$, which completes the proof of part (i). The proofs of the other parts are similar.
- The proof is similar to the proof of part (a).

 □

## Further refinements

Theorem 3 can be refined further for some classes of functions, using exponential convexity (for details see [2] and [3]). First, we use linear functional $\Omega_{1,j}$ define in previous section. Under assumptions of Theorem 3(a), we conclude that, for any odd *n* and for any $j\in\{1,2, \ldots, 5\}$, $\Omega_{1,j}$ acts non-negatively on the class of 2*n*-convex functions.

Further, let us introduce a family of 2*n*-convex functions on $[0,\infty) $ with

31 $$ \varphi_{t}(x) = \textstyle\begin{cases} \frac{x^{t}}{t(t-1)\cdots(t-2n+1)},& t\notin\{0,1,\ldots ,2n-1\}; \\ \frac{x^{j}\ln x}{(-1)^{2n-1-j}j!(2n-1-j)!}, & t=j\in\{0,1,\ldots,2n-1\} . \end{cases} $$

This is indeed a family of 2*n*-convex functions since $\frac {d^{2n}}{dx^{2n}}\varphi_{t}(x)=x^{t-2n}\geq0$.

Since $t\mapsto x^{t-2n}=e^{(t-2n)\ln x}$ is an exponentially convex function, the quadratic form

32 $$ \sum_{i,k=1}^{m}\xi_{i} \xi_{k}\frac{d^{2n}}{dx^{2n}}\varphi_{\frac{p_{i}+p_{k}}{2}}(x) $$

is positively semi-definite. According to Theorem 3(a),

33 $$ \sum_{i,k=1}^{m} \xi_{i}\xi_{k}\Omega_{1,j}\varphi_{\frac{p_{i}+p_{k}}{2}} $$

is also positively semi-definite, for any $m\in\mathbb{N}$, $\xi_{i}\in \mathbb{R}$ and $p_{i}\in\mathbb{R}$, showing the exponential convexity of the mapping $p\mapsto\Omega_{1,j}\varphi_{p}$. Specially, if we take $m=2$ in (33) we see additionally that $p\mapsto\Omega _{1,j}\varphi_{p}$ is also a log-convex mapping, a property that we will need in the next theorem.

### Theorem 10

*Under the assumptions of Theorem *3(a) *the following statements hold*:

(i) *The mapping* $p\mapsto\Omega_{1,j}\varphi_{p}$ *is exponentially convex on* $\mathbb{R}$.
(ii) *For* $p, q, r \in\mathbb{R}$ *such that* $p < q < r$, *we have*
  34 $$ ( \Omega_{1,j}\varphi_{q} ) ^{r-p} \leq ( \Omega _{1,j}\varphi_{p} )^{r-q} ( \Omega_{1,j}\varphi_{r} )^{q-p} $$
  *for* $j=1,2,\ldots, 5$.

### Remark 3

We have outlined the proof of the theorem above. The second part of Theorem 10 is known as the Lyapunov inequality, it follows from log-convexity, and it refines the lower (upper) bound for the action of the functional on the class of functions given in (31). This conclusion is a simple consequence of the fact that exponentially convex mappings are non-negative and if an exponentially convex mapping attains zero value at some point it is zero everywhere (see [6]).

A similar estimation technique can be applied for classes of 2*n*-convex functions given in [6]. Lastly, a similar construction can be made for the linear functionals $\Omega_{2,j}$ and $\Omega_{3,j}$ to obtain the inequalities given in Theorem 10 for these functionals.

## Results and discussion

Steffensen’s inequality first appeared in 1918 and has remained of interest for several mathematicians. This paper is devoted to proving a generalization of Steffensen’s inequality which is related to recent and older developments. We also provide applications of the obtained results. In the primary step we prove a simple lemma which approximates a continuously differentiable function in different forms. By using these approximations, we prove such identities as under the condition of 2*n*-convexity and 2*n*-concavity give a generalization of Steffensen’s inequality and its reverse. Subsequently, we provide applications of the results to the theory of $(2n+1)$-convex functions at a point and exponentially convex functions. We also prove the Lyapunov inequality.

## Conclusion

Several identities related to recent generalization of Steffensen’s inequality have been proved. Under the assumption of 2*n*-convexity and 2*n*-concavity, a generalization of Steffensen’s inequality and its reverse has been obtained from the identities. Applications of the results have been presented of the theory of $(2n+1)$-convex functions at a point and exponentially convex functions.
